# Evaluation of the use of the central physician appointment system and patient satisfaction in a training and research hospital: A cross-sectional study

**DOI:** 10.1097/MD.0000000000045507

**Published:** 2025-10-31

**Authors:** Mehmet Koca, Serdar Deniz, Feyza İnceoğlu

**Affiliations:** a Health Sciences Faculty, Malatya Turgut Özal University, Malatya, Turkey; b Medical School, Malatya Turgut Özal University, Malatya, Turkey.

**Keywords:** central physician appointment system, examination waiting time, health, hospital, patient, patient satisfaction

## Abstract

This study aimed to determine the usage status of the central physician appointment system (CPAS) by patients visiting the outpatient clinic of a tertiary hospital and their level of satisfaction with the healthcare services they receive. The sample of this study consisted of 291 patients who received outpatient services at a tertiary healthcare institution in Malatya. The data collection form consisted of 2 sections. The data collection form consisted of 2 parts. The first section included sociodemographic information, and the second section included the Outpatient Satisfaction Scale. In the present study, 291 people participated, of whom 52.9% (n = 154) were female. The findings showed that 17.5% (n = 51) of the study group had no information about the appointment system. Those who had not yet made an appointment with CPAS (by themselves or anyone else) constituted 23.4% (n = 68) of the sample. In addition, 63.2% (n = 141) of those who had made an appointment at least once before this study had made their appointments online (CPAS). The average Outpatient Satisfaction Scale score of the group was 3.40. Gender and income affected patient satisfaction, but there was no significant correlation with other demographic variables. It was determined that the most satisfied issue was Effective Inspection. The least satisfied issue was the Waiting Time and Consultancy dimension.

## 1. Introduction

At its general assembly in 1977, the World Health Organization set the goal of “Health for All in the 21st Century” to raise the health levels of countries and eliminate inequalities between countries.^[1,2]^ Later, at the Alma Ata Conference in 1978, the principles to be followed to achieve this goal were determined and declared. In line with these developments, health policies guided by the support of international organizations have been attempted in our country, but they have not been sufficiently successful. In 2002, it was decided to make important reforms in health policies under the name of “Health Transformation Program.” Under the title of “Health for All,” the basic targets to be implemented in the field of health have been determined. Among these goals was implementing the e-transformation project in health.^[2]^

Advances in technology, hardware, software, and mobile technologies have significantly affected daily life and have begun to play critical roles in healthcare.^[3]^ Thanks to technological developments, the costs of accessing technology have decreased, and access to technology has become easier.^[4]^ Due to the nature of healthcare, its delivery is continuous. It requires the integration of many services, and internet and information technologies, along with many various technologies, have become even more significant for the health sector.^[5]^

The developing technology has facilitated e-transformation in healthcare and many digital projects have been implemented. In this regard, it is the central physician appointment system (CPAS), which was piloted in 2010 and enables appointments to be made from public health institutions throughout the country in 2012. Appointments from CPAS can be made using Alo182, a website and mobile application channels. Patients can effectively manage their time with the help of the CPAS. In addition, CPAS has been effective in shortening the waiting time before examination and reducing the crowds in front of the polyclinic and inside the hospital. CPAS data have helped plan the effective use of the physician workforce, increase efficiency and quality of health services, and develop health policies.^[6,7]^

Hospitals utilize both queuing and appointment systems in tandem. In the queuing system, patients apply to the relevant unit in the hospital, take a turn for the clinic they want to be examined at, and are examined when their turn arrives. When patient demand for the relevant clinic is high, the waiting time for examination becomes longer, causing congestion. In the appointment system, patients arrive at their chosen clinics shortly before their scheduled appointment times because they have made appointments in advance. This approach reduces patient waiting time and increases patient satisfaction.^[8]^ Studies in the literature report that a well-organized hospital appointment system reduces waiting time and increases patient satisfaction.^[9–11]^

Patient satisfaction is a crucial criterion for evaluating the quality of health services.^[12]^ Additionally, patient satisfaction studies are among the most used outcome measures in the healthcare industry.^[13]^ Patient satisfaction is an important tool in management and planning to measure the quality of care and make the necessary arrangements.^[14]^ In a study conducted in Türkiye, Durmuş and Akbolat found that patient satisfaction, patient trust and patient loyalty variables have a strong positive correlation.^[15]^ Studies have reported that patient satisfaction reduces patient complaints and increases customer loyalty.^[16,17]^

Health services employ experts from various disciplines and expensive technologies in their service delivery structure owing to their unique characteristics. Determining the satisfaction levels of patients, who are consumers of healthcare services, is an important data source for healthcare institutions to improve their service quality continuously. Therefore, it is significant to carry out usage situations and patient satisfaction studies of appointment systems used with technology development to make the necessary improvements.

This study aimed to determine the usage status of the CPAS by patients visiting the outpatient clinic of a tertiary hospital and their level of satisfaction with the healthcare services they receive.

## 2. Materials and methods

### 2.1. Type of research, place, and time of conduct

This cross-sectional study was conducted on patients aged 18 and over and without any mental disabilities who received outpatient clinic services from Malatya Training and Research Hospital between February 1, 2024 and February 29, 2024 and agreed to participate in the present study.

### 2.2. Sample selection and number of sampling

According to the calculation made using the G*power 3.1 program, the sample size was determined to be at least 262, with an effect size of 0.20, margin of error of 0.05, confidence level of 0.95, and representative power of the universe of 0.95, and it was aimed to reach 300 people. The data of 9 patients with incomplete responses were excluded from the analysis and the data of 291 people were included in the present study.

In our study, convenience sampling, one of the non-probability sampling methods, was used. In convenience sampling, it is assumed that individuals within the target population have similar characteristics, i.e., they have a homogeneous structure. According to this assumption, selecting the sample randomly, geographically close, from voluntary participants, or from individuals who are difficult to reach does not create a meaningful difference in the research results.^[18]^ For this reason, convenience sampling was preferred in our study because the sample group was determined spatially in order to reach patients using the CPAS.

### 2.3. Data collection tools

The survey form used in this research consisted of 2 parts. Data were collected through “Socio-Demographic Information” created by the researchers in the first part and “Outpatient Patient Satisfaction Scale” developed by Kaya and Maimaiti in the second part. The applied 5-point Likert type scale consists of 29 items and in scoring the scale responses; 1 = Strongly Disagree, 2 = Disagree, 3 = Undecided, 4 = Agree and 5 = Strongly Agree. The scale consists of 5 sub-dimensions. These dimensions consist of “Appointment, Effective Inspection, Employees Attitude, Waiting Time and Consultancy, General Satisfaction” dimensions. Scores of the total and sub-dimensions of the scale are evaluated as “strongly disagree” between 1 and 1.79 points, “disagree” between 1.80 and 2.59 points, “disagree” between 2.60 and 3.39 points, “undecided” between 3.40 and 4.19 points, “agree” between 3.40 and 4.19 points, and “strongly agree” between 4.20 and 5 points. As the score of the total and sub-dimensions of the scale increases, the perception of patient satisfaction also increases. Cronbach’s α value of the scale was 0.95.^[19]^ In this study, the Cronbach’s α value of the scale was calculated as 0.94.

### 2.4. Statistical analysis

The analysis of the data included in this research was carried out using the SPSS (Statistical Program in Social Sciences) 25.0 program. In the present study, where the significance level (*P*) was taken as .05 for comparison tests, the values of the variables were given as numbers and percentages. Since the data conformed to normal distribution, comparisons were made with the significance test (*t*-test) of the difference between 2 means in independent double groups and with analysis of variance test in independent multiple groups. In the analysis of categorical data, the chi-square (χ^2^) test was applied in comparisons made by creating 2*2 cross tables. “Pearson” correlation coefficient was used to evaluate the relationship between measurement values.

### 2.5. The ethical aspect of this research

Ethical approval for this research was obtained from Malatya Turgut Özal University Non-İnterventional Clinical Ethics Committee with its letter dated 18.12.2023 and numbered E-30785963-020-192086 in Türkiye. The present research was conducted in accordance with the principles of the Declaration of Helsinki.

## 3. Results

The findings in this study showed that 48.1% (n = 140) of the study group resided in the city center. The mean age was 37.20 ± 15.61 (min. 18, max. 82), 52.9% (n = 154) were female, 58.4% (n = 170) were married, 16.5% (n = 48) were civil servants, 10.3% (n = 30) were retired, and 27.5% (n = 80) were at primary school or lower level of education. The total monthly household income of 21.3% of the participants (n = 62) was below the minimum wage (17,002 TL). Those with at least one chronic disease in their family constituted 49.5% (n = 144) of the group (Table 1).

**Table 1:** Sociodemographic characteristics of the study group.

		n	%
Gender	Female	154	52.9
	Male	137	47.1
Age Group	18-29	116	39.9
	30–39	61	21.0
	40–49	39	13.4
	50–59	44	15.1
	60 and older	31	10.7
Residency	City Center	140	48.1
	County	151	51.9
Educational Status	Primary education and below	80	27.5
	High school	102	35.1
	Associate degree and above	109	37.5
Marital status	Married	170	58.4
	Single	121	41.6
Working status	Officer	48	16.5
	Workers. farmers, etc	46	15.8
	Tradesmen, freelancers, etc	31	10.7
	Housewife, unemployed etc	53	18.2
	Retired	30	10.3
	Other	83	28.5
Monthly household income (Turkish Lira)	<17,002 (Below minimum wage)	62	21.3
	Minimum wage (17,002)–20,000	60	20.6
	20,001–25,000	35	12.0
	25,001–30,000	29	10.0
	30,001–40,000	43	14.8
	40,001–50,000	33	11.3
	50,000 and over	29	10.0
Chronic illness in the family	Yes	144	49.5
	No	147	50.5
Daily internet use	<1 h	72	24.7
	1–<2 h	42	14.4
	2–<3 h	67	23.0
	3–<4 h	31	10.7
	≥4 h	79	27.1

17.5% (n = 51) of the study group had no information about the appointment system. Those who had not yet made an appointment with CPAS (by themselves or anyone else) constituted 23.4% (n = 68) of the group. 63.2% (n = 141) of those who had made an appointment at least once so far had made their appointments on the Internet (CPAS). Among the 201 participants who had been to the CPAS at least once without an appointment, 45.8% (n = 92) stated that they could not be examined when they came without an appointment, while 12.4% (n = 25) stated that they could sometimes be examined when they came without an appointment. In addition, 80.1% (n = 233) of the participants said they would recommend the hospital to others (Table 2).

**Table 2:** Characteristics of the study group regarding the use of CPAS.

		n	%
Is there information about CPAS?	Yes	240	82.5
	No	51	17.5
Has a CPAS appointment ever been made for him/her (by him/herself or a relative)?	Yes	223	76.6
	No	68	23.4
Method (usually) used when making an appointment (N = 223)*	182 hotline	82	36.8
	Internet (Through CPAS)	141	63.2
Person making the appointment (N = 223)*	Myself	172	77.1
	I’m close	51	22.9
Would you come to the examination without making an appointment?	Yes	201	69.1
	No	90	30.9
Can you be examined without an appointment? (N = 201)†	Yes	84	41.8
	No	92	45.8
	Sometimes	25	12.4
Would you recommend this hospital to others?	Yes	233	80.1
	No	58	19.9

* Only those who made an appointment were evaluated.

† Only those who came for examination without making an appointment were evaluated.

The average Outpatient Satisfaction Scale score of the group was 3.40 ± 0.75. While the highest mean score (3.62 ± 0.86) was determined from the Effective Inspection sub-dimension, the mean score was determined to be 2.88 ± 1.25 in the Waiting Time and Consultancy sub-dimension, which is the sub-dimension with the lowest satisfaction (Table 3).

**Table 3:** Outpatient satisfaction scale total and sub-dimension mean scores.

Dimensions	Average	Standard deviation	Minimum	Maximum
Scale score	3.40	0.75	1.00	5.00
Appointment sub-dimension	3.37	0.90	1.00	5.00
Active inspection sub-dimension	3.62	0.86	1.00	5.00
Employee attitude sub-dimension	3.40	1.11	1.00	5.00
Waiting time and counseling sub-dimension	2.88	1.25	1.00	5.00
General satisfaction sub-dimension	3.18	1.06	1.00	5.00

There was a positive correlation between the scores of the Outpatient Satisfaction Scale and all subscales (*P* < .01). The strongest correlation (*r*= = 0.912) was between the General Scale Score and the Effective Examination sub-dimension, and the weakest correlation (*r* = 0.205) was between the Appointment sub-dimension and the Waiting Time and Counseling sub-dimension (Table 4).

**Table 4:** Pearson correlation coefficients of outpatient satisfaction scale total and sub-dimension scores.

	Scale score	Appointment sub-dimension	Active inspection sub-dimension	Employee attitude sub-dimension	Waiting time and counseling sub-dimension	General satisfaction sub-dimension
Scale score	1	.587*	.912*	.806*	.661*	.717*
Appointment sub-dimension		1	.418*	.331*	.205*	.330*
Active inspection sub-dimension			1	.708*	.493*	.491*
Employee attitude sub-dimension				1	.568*	.512*
Waiting time and counseling sub-dimension					1	.443*
General satisfaction sub-dimension						1

* *P* < .01.

When the relationship between the scale score and sociodemographic variables was examined, the average score of women (3.27 ± 0.76) was lower than that of men (3.54 ± 0.72) (*P* < .05). The mean score (3.03 ± 0.90) of those whose total monthly income was 50,000 TL and above was significantly lower than those in the other income group (*P* < .05). Age group, place of residence, educational status, marital status, employment status, presence of a chronic disease in the family, and daily internet usage time did not significantly affect the average score (*P* > .05) (Table 5).

**Table 5:** Relationship between sociodemographic characteristics and Outpatient Satisfaction Scale.

		n	Average	Standard deviation	*t/F*	*P*
Gender	Female	154	3.27	.76	3.088	.002
	Male	137	3.54	.72		
Age Group	18–29	116	3.35	.80	0.701	.592
	30–39	61	3.53	.70		
	40–49	39	3.37	.67		
	50–59	44	3.42	.79		
	60 and older	31	3.32	.75		
Residency	City Center	140	3.39	.85	0.235	.814
	County	151	3.41	.66		
Educational Status	Primary education and below	80	3.46	.71	0.383	.682
	High school	102	3.37	.79		
	Associate degree and above	109	3.38	.76		
Marital status	Married	170	3.42	.75	0.462	.644
	Single	121	3.38	.76		
Working status	Officer	48	3.44	.89	1.129	.345
	Workers. Farmers, etc	46	3.57	.70		
	Tradesmen, freelancers, etc	31	3.43	.84		
	Housewife, unemployed etc	53	3.29	.71		
	Retired	30	3.51	.42		
	Other	83	3.30	.78		
Monthly household income (Turkish Lira)	<17,002 (Below minimum wage)	62	3.50	.66	2.926	.009
	Minimum wage (17,002)–20,000	60	3.38	.77		
	20,001–25,000	35	3.57	.68		
	25,001–30,000	29	3.09	.79		
	30,001–40,000	43	3.55	.79		
	40,001–50,000	33	3.46	.61		
	50,000 and over*	29	3.03	.90		
Chronic illness in the family	Yes	144	3.38	.78	0.459	.647
	No	147	3.42	.73		
Daily internet use	<1 h	72	3.36	.79	0.202	.937
	1–<2 h	42	3.45	.57		
	2–<3 h	67	3.40	.76		
	3–<4 h	31	3.34	.67		
	≥4 h	79	3.44	.85		

* Group(s) causing the difference.

In the evaluation made according to the status of using CPAS, it was found that the mean score of those who went to the outpatient clinic and could be examined without an appointment (3.56 ± 0.63) was significantly higher than those who could not be examined without an appointment (3.26 ± 0.70). The mean score of those who stated that they would recommend the hospital to others (3.55 ± 0.65) was significantly higher than those who would not (2.78 ± 0.83) (*P* < .05). Whether there was information about CPAS, whether an appointment had been made before, the method of making an appointment, the person who made the appointment, and whether the patient came to the examination without making an appointment did not significantly affect the scale score (*P* > .05) (Table 6).

**Table 6:** Relationship between the study group’s use of CPAS and the Outpatient Satisfaction Scale.

		n	Average	Standard deviation	*t/F*	*P*
Is there information about CPAS?	Yes	240	3.37	.75	1.699	.090
	No	51	3.56	.78		
Has an CPAS appointment ever been made for him/her (by him/herself or a relative)?	Yes	223	3.37	.72	1.027	.305
	No	68	3.48	.87		
Method used when making an appointment (N = 223)*	182 hotline	82	3.36	.79	0.284	.777
	Internet (Through CPAS)	141	3.38	.68		
Person making the appointment (N = 223)*	Myself	172	3.35	.71	0.767	.444
	I’m close	51	3.44	.75		
Would you come to the examination without making an appointment?	Yes	201	3.42	.72	0.765	.445
	No	90	3.35	.83		
Can you be examined without an appointment? (N = 201)†	Yes‡	84	3.56	.63	4.331	.014
	No	92	3.26	.70		
	Sometimes‡	25	3.54	.93		
Would you recommend this hospital to others?	Yes	233	3.55	.65	7.695	<.001
	No	58	2.78	.83		

* Only those who made an appointment were evaluated.

† Only those who came for examination without making an appointment were evaluated.

‡ Group(s) causing the difference.

## 4. Discussion

Despite the organizational developments in hospitals today, long waiting times may negatively affect patient satisfaction.^[8]^ It increases the institutional loyalty of patients by determining satisfaction levels, increasing service quality and providing more qualified service in line with expectations. Therefore, the satisfaction level of service recipients, which is an important indicator of quality service in the field of health, is also significant for health institutions.^[20]^ Habibi et al in their study examining the effect of appointment planning in an outpatient clinic in Iran on patient satisfaction stated that waiting time has an effect on patient satisfaction. He reported that an appointment scheduling system may be useful in improving patient satisfaction.^[21]^

Yildizbaşi et al stated in their study that the rate of patients with knowledge about CPAS was 83.30 in 2014, 81.55 in 2015, and 83.33 in 2016.^[22]^ In this study, the rate of patients who had knowledge about CPAS was 82.50%. The study results are consistent.

In our research, 77.1% of those who made an appointment with CPAS were able to make an appointment for themselves. In his study conducted in Istanbul, Kağan (2014) found that the rate of those who made their own appointments from CPAS was 75.1%.^[23]^ The findings suggest that people who do not make their own appointments have a low level of education, a high level of age, and inadequate use of technological devices.

In our study, the rate of those who made an appointment with CPAS at least once was 76.6%. Yildizbaşi et al found in their study that the rate of those who made an appointment with CPAS at least once was 83.33% in 2014, 74.04% in 2015, and 80% in 2016.^[22]^ Although the results are close to each other, when this result is considered together with those who have knowledge about CPAS, it is seen that the awareness and use of CPAS have not increased much over the years since its introduction.

The Ministry of Health stated that the rate of those who made appointments using mobile application and internet from CPAS in 2016 was 31%, and in 2023 this rate was 37.3%.^[6,24]^ In his study, Kağan found that the rate of patients making CPAS appointments using internet and mobile application was 46.37%.^[23]^ In a study conducted by Kurşun and Kaygisiz, 29.71% of patients made their appointments through the website.^[25]^ Kiraç’s study showed that 49% of patients made their appointments online or through mobile applications.^[26]^ In this study, the rate of patients who made an appointment from CPAS using the internet and mobile application was 63.2%. Making a CPAS appointment using the internet or mobile application is important in terms of reducing the workload on the Alo 182 line and using the system effectively and efficiently.

In a study conducted in China, it was reported that those who used the web-based appointment system had a higher satisfaction rate than those who used the regular queuing system. In the same study, the reasons for not using the web-based appointment system were listed as being unaware of the web-based system, not trusting the internet, and insufficient computer usage skills.^[27]^ In another study conducted in China, the rate of those using internet-based appointment systems was 17%.^[28]^ In line with these results, it would be beneficial to reduce unnecessary waiting in the hospital and increase the effective use of the physician workforce, increase awareness of CPAS and make appointments through the system, and also increase the level of technological literacy to increase patient and employee satisfaction and patient safety.

In their study on 531 patients in Konya, Kaya and Maimati found the overall score of the scale to be 3.55. It was determined as 3.34 in the Appointment sub-dimension, 3.62 in the Employees’ Attitude sub-dimension, 3.84 in the Meat Inspection sub-dimension, 2.80 in the Waiting Time and Consultancy sub-dimension, and 3.43 in the General Satisfaction sub-dimension.^[19]^ In Atay’s study using the same scale on 384 patients receiving outpatient clinic services in Samsun, the Scale General score was 3.54. In the same study, it was 3.65 in the Appointment sub-dimension, 3.69 in the Effective Inspection Dimension, 3.48 in the Employee Attitude Dimension, 2.86 in the Waiting Time and Consultancy Dimension, and 3.50 in the General Satisfaction Dimension.^[29]^

In this study, the patient satisfaction Scale General score was 3.40. In the sub-dimensions of the scale, it was 3.37 in the Appointment dimension, 3.62 in the Effective Inspection dimension, 3.40 in the Employee Attitude dimension, 2.88 in the Waiting Time and Consultancy dimension, and 3.18 in the General Satisfaction dimension. Similarly, according to the results of this study, the highest score average was 3.62 in the Effective Inspection sub-dimension, while the average score in the Waiting Time and Consultancy sub-dimension, which is the sub-dimension with the lowest satisfaction, was 2.88. As a result of their studies, Kaya and Maimati and Atay found that the satisfaction scale found to be similar regarding the dimensions with the lowest and highest scores. This similarity can be interpreted to mean that effective examination has an important place in patient satisfaction and that an increase in the waiting time for examination significantly decreases satisfaction.

In a study conducted in Iran, Zarei stated that the dimension that outpatients were least satisfied with was the dimension related to waiting time.^[30]^ Ganasegeran et al in their study in Malazeya found that the dimension with the highest score was the technical quality dimension of the quality of service received by the patient.^[31]^ It is seen that the highest and lowest score dimensions obtained in the study are consistent with the results of some studies in the literature.

Ferreira et al listed the most frequently used patient satisfaction criteria through systematic literature and bibliometric analysis and determined that waiting time was the second most used variable according to this list. In the same study, waiting time ranked second after medical care among the most critical factors for patient satisfaction.^[32]^ McMullen and Netland reported that time spent waiting in the queue, knowledge of the doctor and time spent with the doctor had the highest impact on overall satisfaction.^[33]^ In this study, the strongest correlation (*R* = 0.912) was between the General Scale Score and the Effective Examination sub-dimension. This result and other studies in the literature reveal that waiting time and effective examination are crucial for patient satisfaction in health service delivery. To increase patient satisfaction, which is frequently used in the evaluation of the provision of health services, it is necessary to manage the time of patients and health personnel. In this respect, increasing the effective use of CPAS in Türkiye plays a critical role in patient satisfaction.

In our study, no significant relationship was found between marital status and occupation and satisfaction. This finding is consistent with the findings of some studies in the literature.^[29,34]^

Polat and Atay stated in their study that there is no significant relationship between age and education level and patient satisfaction.^[29,35]^ However, Şantaş et al found a significant relationship between age and educational status and patient satisfaction in their study.^[34]^ In this study, no significant relationship was found between age and educational status and patient satisfaction. While this result is compatible with the results of Polat and Atay, it is different from the results of Şantaş et al.^[29,34,35]^ It is stated that increasing the level of education may decrease satisfaction as it increases expectations.^[19,34]^ The difference in research results may be due to the difference between the research design and the sample group.

The rate of those who came to the hospital without an appointment was 69.1% and 41.8% of those who came to the hospital without an appointment stated that they could be examined without an appointment. It was also determined that there was a significant relationship between those who were examined at the hospital without an appointment and patient satisfaction. The high satisfaction rate in these patients is related to the patient’s expectations, and since being examined without an appointment is a privilege granted to the patient, this situation increases the positive impression of the health institution and its staff in patients and this is reflected in the satisfaction status. Similarly, a significant relationship was found between satisfaction and patients recommending the hospital to others. Consistent with this finding, the positive image of the hospital where the patient is examined has an effect on patient satisfaction. In some studies in the literature, it is stated that the satisfaction result of those who recommend the institution to others is higher.^[29,36]^

Thornton et al stated that there was a significant relationship between patient satisfaction and residence in their study conducted in the United States of America. They found that patients living in the city center were more satisfied than patients living in rural areas.^[37]^ However, in our study, it was determined that place of residence had no effect on patient satisfaction. This difference is due to transportation differences between the places where the present research was conducted.

## 5. Conclusion

As a result of the present study, the rate of those who have knowledge about the CPAS is 82.5%, the rate of those who have made an appointment with CPAS at least once is 76.6%, and 63.2% of these appointments have generally made an appointment using the internet or mobile application and have made an appointment with CPAS. It was concluded that 77.1% of those who made an appointment made their own appointments. It was also determined that 69.1% of the participants went to the hospital to be examined even if they did not have an appointment, 41.8% of those who went were able to be examined, and 12.4% were sometimes able to be examined.

In this research, it was concluded that patient satisfaction was affected by gender and income and that there was no significant relationship between it and other demographic variables. The average of the patient satisfaction scale was 3.40, and the most satisfied subject in the sub-dimensions was Effective Examination. The least satisfied subject was the Waiting Time and Consultancy dimension.

For health institutions to plan their workforce correctly and reduce the waiting time of patients, patients should be encouraged to make an appointment with CPAS before the examination. Given that unnecessary hospital applications are among the biggest reasons for waiting times and inability to be examined, the behavior of applying to the right health institution at the right time should be introduced to society. Public service announcements should be frequently used to ensure awareness of the methods and advantages of making an appointment simultaneously.

## Author contributions

**Conceptualization:** Mehmet Koca, Serdar Deniz, Feyza İnceoğlu.

**Data curation:** Mehmet Koca, Serdar Deniz, Feyza İnceoğlu.

**Formal analysis:** Mehmet Koca, Serdar Deniz, Feyza İnceoğlu.

**Investigation:** Mehmet Koca, Serdar Deniz, Feyza İnceoğlu.

**Methodology:** Mehmet Koca, Serdar Deniz, Feyza İnceoğlu.

**Project administration:** Mehmet Koca, Serdar Deniz, Feyza İnceoğlu.

**Resources:** Mehmet Koca, Serdar Deniz, Feyza İnceoğlu.

**Software:** Mehmet Koca, Serdar Deniz, Feyza İnceoğlu.

**Supervision:** Mehmet Koca, Serdar Deniz, Feyza İnceoğlu.

**Validation:** Mehmet Koca, Serdar Deniz, Feyza İnceoğlu.

**Visualization:** Mehmet Koca, Serdar Deniz, Feyza İnceoğlu.

**Writing – original draft:** Mehmet Koca, Serdar Deniz, Feyza İnceoğlu.

**Writing – review & editing:** Mehmet Koca, Serdar Deniz, Feyza İnceoğlu.
